# Forensic Characteristics of Physical Elder Abuse and Current Status and Issues of Collaboration between Forensic Medicine Departments and Related Institutions in Japan

**DOI:** 10.3390/ijerph192215382

**Published:** 2022-11-21

**Authors:** Maiko Toya, Saki Minegishi, Hajime Utsuno, Jun Ohta, Shuuji Namiki, Kana Unuma, Koichi Uemura, Koichi Sakurada

**Affiliations:** 1 Department of Forensic Dentistry, Graduate School of Medical and Dental Sciences, Tokyo Medical and Dental University, 1-5-45 Yushima, Bunkyo-ku, Tokyo 113-8510, Japan; 2 Department of Forensic Medicine, Graduate School of Medical and Dental Sciences, Tokyo Medical and Dental University, 1-5-45 Yushima, Bunkyo-ku, Tokyo 113-8519, Japan

**Keywords:** autopsy, elder abuse, elder mistreatment, elderly, physical abuse

## Abstract

This study sought to clarify the characteristics and trends of physical elder abuse and the status of collaboration between forensic medicine departments and related institutions in Japan. Questionnaires were sent to 82 forensic medicine departments and 2857 institutions randomly selected from hospitals, municipalities and public community general support centers. The survey period was February to June 2021, including an extension period for collection. Responses from 675 facilities were analyzed. The most common finding in cases of physical elder abuse at forensic medicine departments was subcutaneous hemorrhage on the head (85.7%), with mixed old and new injuries most commonly observed in the lower limbs (70%). There were few cases in which there was collaboration between forensic medicine departments and other institutions. Among the issues identified, there is a need to provide related institutions with information obtained in forensic medicine departments. A new collaboration system is needed to achieve this.

## 1. Introduction

Elder abuse was first reported as “granny battering” in the 1970s [1]. More than three decades later, the prevalence of elder abuse was reported to be high in the European community [2], 1 in 10 respondents in a US study reported some type of elder abuse [3] and approximately 11% of the elderly in India have experienced some type of abuse [4]. Globally, elder abuse is estimated to affect around one in six elderly people [5]. According to recent reports, the prevalence of elder abuse has increased further during the COVID-19 pandemic [6,7,8]; the current pandemic may impact older people and their caregivers [9], making elder abuse a major social issue worldwide, and ways to moderate the risks have been suggested [10]. In 2021, the World Health Organization defined elder abuse as “a single or repeated act, or lack of appropriate action, occurring within any relationship where there is an expectation of trust, which causes harm or distress to an older person” [11].

In Japan, the elderly population is defined as people aged 65 years or older [12], and elder abuse is classified into physical abuse, psychological abuse, neglect, sexual abuse and financial abuse under Article 2 of the Elder Abuse Protection Law, enacted in 2006. Similar classifications of elder abuse are used in many countries [13]. The number of cases of elder abuse remains high. In FY2019, the Ministry of Health, Labour and Welfare reported 644 cases of elder abuse by care facility staff and 16,928 cases by caregivers, with the number of consultation/reports exceeding the previous record [14]. Factors in abuse have gradually become clear [15], yet fatal cases of abuse continue to be reported year after year. In FY2019, there were 15 fatal cases of elder abuse, most commonly involving physical abuse [14]. Given this situation, deaths and serious cases reported by government have since been reanalyzed [16], however, few studies have examined the characteristics of injuries to clarify signs of elder physical abuse in fatal cases. Collaboration between related institutions handling elder abuse cases is important for identifying abuse early and providing support promptly, yet the status of collaboration and associated problems and solutions have not yet been investigated in Japan. In fact, considerable information about physical abuse can be obtained through forensic autopsies. In Japan, the most common cause of death reported in forensic autopsy cases of elder abuse is subdural hemorrhage [17]; however, the forensic characteristics of injuries and trends have not been studied in detail. In regards to child abuse, on the other hand, progress has been made based on approaches taken in forensic medicine [18] and through collaboration between forensic medicine and municipalities [19]. In regards to elder abuse, however, it is clear that a sufficient intervention system has not been established. The government has reported only a 50% implementation rate in efforts to build a network of intervention support for related organizations [14].

Despite the fact that elder abuse is a worldwide public health issue, it is not well researched and the number of studies conducted varies from country to country [20]. Outside Japan, a forensic study of elder abuse cases by the Brazilian Institute of Forensic Medicine investigated the characteristic of physical abuse of elderly persons (age 60 years or older) who underwent forensic examinations and reported on the attributes of the abuser and the abused [21]. In the United States, the Adult Protective Services (APS) agency plays an important role in intervening in cases of elder abuse, and according to Lachs and Pillemer [22], interventions for identified cases are not only tailored to the victim’s situation, but also rely heavily on local resources and on family resources and dynamics. Some regions operate forensic centers staffed by multidisciplinary teams that include experts in law, criminal justice, medicine and social welfare [23]. These centers’ activities are attracting attention and expanding [24], but no details on the actual state of collaboration between related institutions, including forensic medicine departments, have been reported to date. We believe it would be beneficial to many countries to know how related institutions that deal with elder abuse and various compounding problems collaborate in other countries, as well as to understand the issues they face in collaborating and the solutions attempted, even though the systems for collaboration will be different.

Questionnaire surveys of forensic medicine departments should be able to provide useful information that could help reduce the number of cases of elder abuse. Therefore, in this study, we conducted a questionnaire survey of forensic medicine departments and related institutions in Japan with the aim of clarifying characteristics and trends in physical elder abuse. We analyzed and compared injuries in autopsy cases at forensic medicine departments and in injury cases at related institutions in order to understand the status of collaboration between them and identify methods for effective collaboration to prevent elder abuse in Japan. The findings are also expected to provide useful information for countries that operate different systems.

## 2. Materials and Methods

### 2.1. Selection of Target Facilities

This study targeted 82 forensic medicine departments at universities, 953 hospitals, 1000 municipalities and 904 public community general support centers in Japan. Because the Elder Abuse Protection Law stipulates that municipalities mainly handle cases of elder abuse in collaboration with public community general support centers, we excluded from the survey public community general support centers with the same address as municipal offices, university hospitals with a forensic medicine department and children’s hospitals. The questionnaire was mailed to their facilities.

This study was approved by the research ethics committee of our institution and conformed to the provisions of the Declaration of Helsinki.

### 2.2. Data Collection and Survey Period

Self-administered questionnaires with the name of the facility were sent by mail with a request to complete the questionnaire and return it using a stamped-addressed envelope. The survey sheets, invitation letter, informed consent form and stamped-addressed envelope were sent to hospital administrators and the persons in charge at forensic medicine departments, relevant departments in municipalities and public community general support centers. The survey period was February to June 2021, including an extension period for collection. The questionnaire items for each facility were as follows.

Forensic medicine departments: name of the department; presence of an enquiries desk for consultations and examinations on elder abuse; autopsy cases involving elder abuse in FY2017-FY2019 (number of cases, type of autopsy, sex and age of cases, type of abuse, perpetrator, location of abuse, means of abuse, cause of death based on autopsy findings, inconclusive cases of elder abuse and reasons); cases of elder abuse other than autopsy cases (number of cases, sex and age of cases, type of abuse, perpetrator, location of abuse, means of abuse, reporter of abuse and details, areas requested for examination, reason for request, location where direct examination of living patients was performed, factors considered in direct examination of living patients, materials used for indirect examination of living patients, method for communicating the examination results to the reporter of abuse); injuries (type and site of injury, characteristics of injuries caused by elder abuse, site of mixtures of old and new injuries); collaboration with hospitals, municipalities or public community general support centers (things that became possible through collaboration, requirements for strengthening collaboration, the need for other institutions to easily contact departments of forensic medicine, trends in elder abuse, thoughts about the involvement of forensic medicine in elder abuse).

Hospitals: name of the facility; presence of an enquiries desk for consultation and examinations on elder abuse; cases of elder abuse in FY2019; details of cases (number of cases, sex and age of cases, type of abuse, perpetrator, location of abuse, means of abuse, reporter of abuse, background to detecting the abuse, cases reported to the municipality or public community general support center and feedback on the case, departments that provided support, cases where judging whether abuse occurred was difficult, trends in elder abuse); injuries (findings, photos of injuries); collaboration with municipalities or public community general support centers (number of cases, details of collaboration, effects of cooperation, requirements for strengthening collaboration); collaboration with forensic practitioners or forensic medicine departments (number of cases, requirements for strengthening collaboration, reasons for lack of collaboration, things needed for future collaboration).

Municipalities and public community general support centers: name of the facility, presence of an enquiries desk for consultation and examinations on elder abuse; cases of elder abuse in FY2019; details of cases (number of cases, sex and age of cases, type of abuse, perpetrator, location of abuse, means of abuse, reporter of abuse, cases where judging whether abuse occurred was difficult, trends in elder abuse, cases of temporary separation, cases where a decision on temporary separation could not be made and reasons, trends in elder abuse, ex-post evaluations); injuries (findings, photos of injuries); collaboration with hospitals (number of cases, details of collaboration, effects of collaboration, requirements for strengthening collaboration); collaboration with forensic practitioners or forensic medicine departments (number of cases, requirements for strengthening collaboration, reasons for lack of collaboration, things needed for future collaboration).

### 2.3. Statistical Analysis

For analysis, simple tabulation of basic data and responses to questionnaire items was carried out and calculations were performed using SPSS (IBM SPSS Statistics for Windows, version 28.0). Fisher’s exact test was used to determine differences in injuries reported between forensic medical departments and related institutions, and statistical significance was set at a *p*-value of <0.05.

## 3. Results

### 3.1. Responses from Forensic Medicine Departments

The response rate from forensic medicine departments was 41.5% (34 departments), and two departments did not respond to the item about having an enquiries desk for consultation and examinations for elder abuse. Of the remaining 32 departments, four (12.5%) answered that they have an enquiries desk; for example, “We have an enquiries desk for child abuse, but we also handle enquiries for elder abuse” and “We deal with enquiries face to face”.

#### 3.1.1. Autopsy Cases

**Figure 1:**
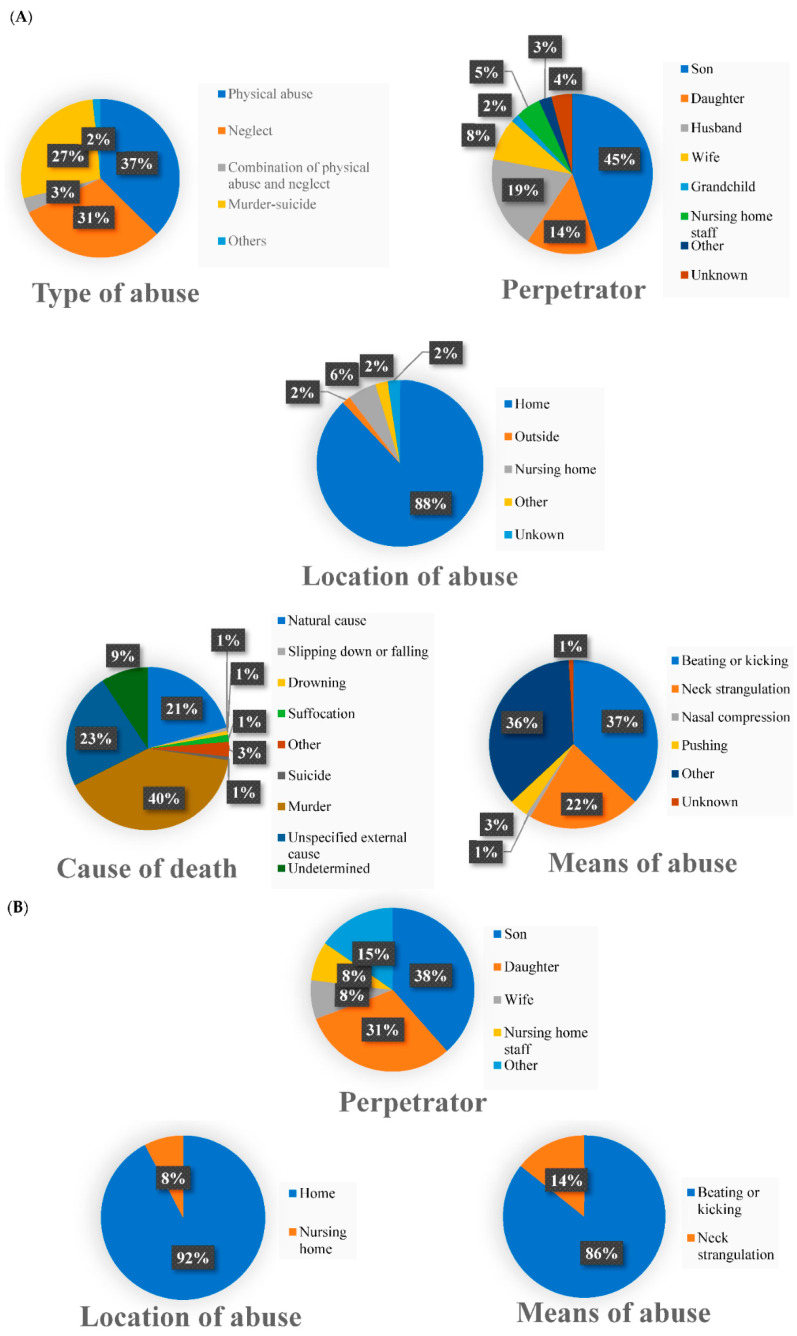
Details of cases of elder abuse reported by forensic medicine departments. (**A**) Autopsy cases; type of abuse, perpetrator, location of abuse, means of abuse, and cause of death. (**B**) Cases of elder abuse other than autopsy cases; perpetrator, location of abuse, and means of abuse.

Eighteen of 33 forensic medicine departments (54.5%) had autopsy cases involving elder abuse. One department did not answer this question. There were 41 cases in FY2017, 44 in FY2018 and 51 in FY2019. Victims were 47 men and 89 women. Of the victims, 50% were in their 80s and 30% were in their 70s. Types of abuse were physical abuse in 37% of cases, neglect in 31% and murder–suicide in 27%. The most common perpetrator was the victim’s son (45%), followed by husband (19%) and daughter (14%). The most common site of elder abuse was the victim’s home (110 cases); other locations listed were a car and a storage facility. The most common means of abuse was beating or kicking (41 cases, 37%), followed by neck strangulation (24 cases, 22%). Causes of death based on autopsy findings were as follows: murder, 56 cases (40%); other or unspecified external cause, 32 cases (23%); natural causes, 29 cases (21%); undetermined, 13 cases (9%); other, four cases (3%); suffocation, two cases (1%); and one case each (1%) of slipping down or falling, drowning, and suicide. Type of abuse, perpetrator, location of abuse, means of abuse and cause of death are summarized in Figure 1.

Eleven of 17 forensic medicine departments (64.7%) had cases with an inconclusive relationship between cause of death and elder abuse based on autopsy findings. Seventeen departments did not answer this question. Details of reasons for inconclusive results were as follows: malignant tumor/senile decay; possibility of accidental death by falling; cause of death undetermined, putrefaction, advanced postmortem changes with no relation to fatal injury, no fatal injury found, making the cause of death difficult to determine; injuries due to abuse that was mild or old and injuries had healed.

#### 3.1.2. Cases of Elder Abuse Other Than Autopsy Cases

Five forensic medicine departments (14.7%) reported handling cases of elder abuse other than autopsy cases, with three cases reported in FY2017, four cases in FY2018 and six cases in FY2019. Victims were six men and seven women. Of the victims, 54% were in their 80s and 38% were in their 70s. All types were physical abuse. The most common perpetrator was the victim’s son (38%), followed by the daughter (31%). The most common location of elder abuse was the victim’s home (92%). The most common means of abuse was beating or kicking (86%), followed by neck strangulation (14%). Perpetrator, location of abuse and means of abuse are summarized in Figure 1.

The reporter of abuse was from a municipality in one case and the police in two cases. Reasons for requesting an expert opinion were difficulty in judging whether an injury was due to accident or abuse (33.3% of cases), mechanism of injury was unknown (25%), time of injury was unknown (25%) and difficulty in deciding from the overall assessment whether abuse was occurring (16.7%). Responses about the location where direct examination of living patients were performed were one case each of the victim’s home or nursing home, depending on the case, and other were one case each. Answers about the factors considered in the direct examinations of living patients were, in one case each, performing the examination with relevant staff present, coping with the victim’s dementia, being careful not to scare the victim, trying to ask objective, non-leading questions, making a house call because the victim was old and had difficulty moving, taking proper photos and performing the examination as usual. The materials used for indirect examination of living patients were photos (100%), X-rays (75%), case records (50%) and blood test results (25%). The methods for communicating the examination results to the reporter of the abuse were documents (75%), e-mail (25%) and other (25%).

#### 3.1.3. Injuries in Victims of Elder Abuse

Injuries reported by the 14 forensic medicine departments that answered this question are shown in Table 1. The most common type and site of injury was subcutaneous hemorrhage on the head (85.7%); other frequent sites of injury were the head, neck, back, and upper extremities. Subcutaneous hemorrhage and abrasions were the most common injuries. Mixtures of old and new injuries occurred most frequently on the lower extremities (70%), followed by the head (60%), chest or abdomen (60%), upper extremities (60%), back (40%) and neck (30%) (multiple answers allowed).

#### 3.1.4. Collaboration with Hospitals

Four of the 32 forensic medicine departments (12.5%) that responded to this question reported collaboration with hospitals; however, 28 departments did not engage in such collaboration. One case involved providing a lecture and another involved giving a second opinion. Responses about things enabled or possibly enabled by collaborating, requirements for strengthening collaboration, and things needed for other institutions to easily contact forensic medicine departments are shown in Table 2.

#### 3.1.5. Collaboration with Municipalities or Public Community General Support Centers

Two of the 32 forensic medicine departments (6.3%) that answered this question reported collaborating with municipalities or public community general support centers; however, 30 departments did not engage in such collaboration. One case involved providing a lecture and one case was classified as other. Responses about things enabled or possibly enabled by collaborating, requirements for strengthening collaboration, and thing needed for other institutions to easily contact forensic medicine departments are shown in Table 2.

The opinions shared included the following: needing to be aware that the person in charge of the municipality is responsible for responding to elder abuse; not knowing the current needs of the municipality; working with the municipality to inform people about the presence of enquiries desks for handling elder abuse in forensic medicine departments, discussing budget acquisition in the municipality and having regular opportunities to exchange opinions about child abuse; the municipality should also play a role in securing human resources to liaise with related institutions—however, collaborating with the municipality would also mean cutting down on the time spent on research, education and management in parallel, so the notion of making improvements within existing resources in forensic medicine is limited; not knowing what improvements could be made because it was unclear what kind of people in the municipality they would be working with; needing to exchange opinions between forensic practitioners and municipalities face to face, clarifying who the person in charge was and the role of the municipality in elder abuse, in order to establish a system for collaboration; and making it easy for related institutions to ask for help via individual consultations, which is similar to building relationships for easy consultation, although recognizing there are limits to it.

#### 3.1.6. Trends in Elder Abuse

The most commonly reported trends seen in forensic medicine departments were classified as “other” and various responses were received. Some respondents did not notice any trends or did not have enough cases to determine trends, while others noticed some slight or obvious trends related to murder–suicide or injury and neglect. Others gave opinions about trends as follows: elder abuse has happened for a long time and the number of cases has increased with the aging of society; in many cases with advanced postmortem changes due to delayed discovery of death, it was hard to judge whether neglect occurred; information about possible cases of elder abuse were shared in conferences; the number increased of suspicious cases of solitary death involving the elderly who lived alone, so we expect a greater increase in cases of social neglect than in cases of elder abuse; and findings reflect long-term chronic conditions because the elderly are getting older, so the basis for judging whether abuse occurred is very unclear. Among the responses to the item about the involvement of forensic medicine in elder abuse, 74.2% felt that judgements should be made by integrating information obtained from the examinations results in forensics with information obtained from municipalities or public community general support centers, and 61.3% felt that finding and preventing elder abuse requires the involvement of forensic medicine.

### 3.2. Responses from Related Institutions

#### 3.2.1. Responses from Hospitals

The response rate was 15.2% (145 responses). Twenty-six of the 143 hospitals (17.9%) that answered this question had measures in place for handling elder abuse, but 117 hospitals (80.7%) did not, and two hospitals (1.4%) did not answer whether they had it. Details are shown in Table 3. Injuries seen in cases of elder abuse were bruises (76.2%), scratches or wounds (57.1%), fractures (23.8%) and burns (4.8%). None of the institutions collaborated with forensic medicine departments (or forensic practitioners) in FY2019. Reasons for this can be seen in the responses about ways to strengthen future collaboration and things needed for future collaboration (Table 4). Building relationships for easy consultation was the most common thing needed for future collaboration with forensic practitioners (66.1%). In terms of extent of current collaboration with municipalities and public community general support centers, 87.4% of hospitals had not collaborated in any cases, 10.5% had collaborated in 1–3 cases and 2.1% had collaborated in 4–10 cases. The most common type of collaboration was sharing information about the victim’s disease status (57.9%), followed by managing the relationship between the abuser and the victim, family and relatives (52.6%). The responses about the effects of collaborating with municipalities and public community general support centers are shown in Table 4, along with responses about improvements for strengthening collaboration.

#### 3.2.2. Responses from Municipalities

The response rate was 24.3% (243 responses). Overall, 81.5% of municipalities had at least one case of elder abuse and 18.5% had no cases in FY2019. Details are shown in Table 3. Injuries due to elder abuse were bruises (90.7%), scratches or wounds (52.5%), fractures (15.4%) and burns (4.9%). Compared with injuries reported in the forensic medicine departments, there were significantly fewer cases of burns (*p* < 0.05) reported by the municipalities.

None of the municipalities cooperated with forensic medicine departments (including forensic practitioners) in FY2019. Reasons for this can be seen in the responses about the improvements needed to strengthen future collaboration and things needed for future collaboration (Table 4). Building relationships for easy consultation was the most common thing needed for future collaboration with forensic practitioners (64.2%). In terms of extent of current collaboration with hospitals,54.8% had not collaborated in any cases, 37.5% had collaborated in 1–3 cases, 6.3% had collaborated in 4–10 cases and 3.2% had collaborated in more than 10 cases. The most common type of collaboration was sharing information about the victim’s disease status (67.3%).

The responses about the effects of collaborating with hospitals are shown in Table 4, along with responses about improvements for strengthening collaboration.

#### 3.2.3. Responses from Public Community General Support Centers

The response rate was 28.0% (253 responses). Details are shown in Table 3. Injuries due to elder abuse were bruises (90.3%), scratches or wounds (48.9%), fractures (10.8%) and burns (2.8%). None of the institutions cooperated with forensic medicine departments (including forensic practitioners) in FY2019. Reasons for this can be seen in responses about the improvements needed to strengthen future collaboration and things needed for future collaboration (Table 4). Again, building relationships for easy consultation was the most common thing needed for future collaboration with forensic practitioners (61.2%). In terms of the responses about cooperation with hospitals, 62.2% had not collaborated in any cases, 33.7% had collaborated in 1–3 cases and 4.1% had collaborated in 4–10 cases. The most common type of collaboration was sharing information about the victim’s disease status (62%).

The responses about the effects of collaborating with hospitals are shown in Table 4, along with responses about improvements for strengthening collaboration.

When we compared injuries reported between the related institutions, public community general support centers reported significantly fewer fractures than forensic medicine departments (*p* < 0.001) and hospitals (*p* < 0.05) and significantly fewer cases of burns than forensic medicine departments (*p* < 0.05).

## 4. Discussion

### 4.1. Forensic Characteristics of Physical Elder Abuse

Head injuries were found to be particularly prominent in severe cases of physical elder abuse overseas [25], and an autopsy study found that subdural hemorrhage was the most common cause of death in autopsy cases, with subcutaneous hemorrhages found on the head, face and body [17]. Therefore, head injury may be a characteristic of serious cases. In our study, neck strangulation was a frequent means of abuse found at autopsy and subcutaneous hemorrhage in the neck was frequently found, so neck injury was another characteristic of serious cases. At related institutions, external bruises and scratches or wounds were seen in many cases, with most of these injuries noticed by staff during home visits or clinical examinations. There were different trends in abuse between autopsy cases at forensic medicine departments and cases at related institutions, and mild cases seemed to be more common at related institutions. In autopsy cases, the chest or abdomen and sites that had mixtures of old and new injuries often indicated repeated abuse, but depending on the site, injuries might be hidden by clothes and overlooked. Injuries have been reported to less commonly involve the lower extremities than the head, neck and upper extremities [26]. However, in our study, some sites were subjected to repeated abuse, suggesting that coexisting old and new injuries can be useful for judging whether abuse has occurred. Compared with cases at related institutions, fracture was significantly more common in autopsy cases than in cases in municipalities or public community general support centers. Fractures may be hard to detect externally, so careful attention may be needed to detect them in vulnerable elderly people. Although severe cases were among the cases reported by hospitals, many cases at municipalities and public community general support centers seemed to be mild. In mild cases of abuse, injuries due to repeated abuse and findings that could suggest serious outcomes should not be overlooked. Age-related changes can be easily confused with elder abuse [25], so careful judgement is needed to determine whether abuse is present. Rosen et al. [27] reported that, compared with accidental fallers, abuse victims had more maxillofacial, dental and neck injuries. Assessment of injuries at these sites should be given particular attention.

Linking the cause of death to elder abuse is sometimes difficult in forensic medicine departments. Currently, police intervention is common, but direct communication between forensic medicine departments and related institutions seems to be uncommon. Information from forensic autopsy is usually kept confidential as part of criminal investigations. For information sharing, at the very least a system is needed in which forensic medicine departments can directly obtain information from related organizations. In the United States, many institutions deal with elder abuse, and it was reported that doctors play an important role that requires them to be familiar with resources and to coordinate care [22]. In Japan, understanding the role of relevant institutions, including forensic medicine departments, and where to connect cases seems to be important for dealing with elder abuse. As in cases overseas, multidisciplinary teams that include experts in law, medicine and social services [23] might be able to tackle complex problems including family relationships in a comprehensive manner.

### 4.2. Current Status and Issues of Collaboration between Forensic Medicine Departments and Related Institutions

Few cases of collaboration between forensic medicine departments and related institutions were evident in this study. However, there are reports abroad that forensic analysis played an important role in elder abuse cases. Based on analysis of a case that was initially thought to be a natural death but in which neglect was shown to be a contributing factor to the cause of death, Ventura, Caputo, and Molinelli [28] point out that forensic pathologists should conduct thorough postmortem examinations and establish whether abuse is the cause of death. Another study stresses the need for forensic pathologists to recognize elder abuse and neglect in cases where physical abuse and neglect was a significant cause of death [29]. For early detection of elder abuse, it is important to include the specialized knowledge of forensic doctors and nurses in multidisciplinary team consultations [30,31]. In our study, there were a variety of opinions about the number of cases handled to be able to determine trends in elder abuse; however, many respondents discussed assessing from both clinical and forensic perspectives, considering both the results of the forensic examination and the various information known to the municipality when deciding whether abuse has occurred or not and the need for the involvement of forensic medicine in the detection and prevention of elder abuse. It was thought that considering the background to and history of abuse when making a comprehensive decision would help prevent missed decisions. On the other hand, many related institutions responded that they did not understand how forensic medicine was involved in elder abuse cases and that there was no forensic practitioner nearby. In addition, public awareness activities about assessments for elder abuse are needed, but how to communicate its importance was proving to be a challenge. Explaining our findings about the type of injuries commonly seen in victims of elder abuse and in cases of non-autopsy cases seen in related institutions may be especially helpful, even though the number of cases was relatively small. There is also a need for information on the role of forensic medicine and for the free exchange of opinions. However, because securing funding for assessments and human resources in forensics is an issue, cooperation in administrative matters is crucial. Forensic centers in the United States have been established with grants from foundations and the salaries of specialized coordinators are paid with grants, but there is no additional reward for the staff members of the team, and their salaries are limited to their normal work [32]. A similar issue can be seen in the need to secure funding for activities. Federal funding has been reported to promote the spread of multidisciplinary teams [24], so financial support may be necessary to strengthen collaboration in the prevention of elder abuse in Japan as well.

A study overseas found that one in 24 cases of elder abuse was reported to authorities [33]. In our study, there were more autopsy cases involving elder abuse than cases reported by the government from FY2017 to 2019 (28 cases in FY2017, 21 cases in FY2018 and 15 cases in FY2019), suggesting that more cases could exist. In Japan, the Child Abuse Prevention Law stipulates in Article 4, paragraph 5 that the national or local government shall conduct research and verification on matters necessary for the prevention of child abuse. However, the Elder Abuse Protection Law does not require verification. Oppata [34] recommended revision of this law and noted the need to mandate investigation in death or serious cases under Article 26. As in child death reviews for suspected child abuse [35], forensic medicine will become increasingly important in elder abuse.

As a discussion regarding the law in Japan, although reporting of elder abuse is obliged to make efforts except in the case of serious danger, a proposal has been made to revise reporting to make it mandatory by Japan’s Academy for the Prevention of Elder Abuse [36]. It may be useful in preventing abuse in the future.

In Japan, municipalities are the responsible entity in The Elder Abuse Protection Law and outsource some of their work dealing with elder abuse cases to public community general support centers, with social workers and care managers at the center of the response. The social work process is generally divided into intake, assessment, intervention and reassessment in elder abuse cases [37], and considerable information about victims and abusers can be acquired through this process. However, a rationale for intervention and support based on The Elder Abuse Protection Law is currently needed, so in fact intervention and support may not proceed promptly. In these circumstances, Oppata [34] points out the importance of social work practice for elder abuse and states that a precautionary approach is needed, while also highlighting that not needing verification of death by law serves to limit the expansion of social work practice. Collaboration with forensic medicine can help to counteract institutional limitations such as these.

The American Bar Association (ABA) promotes the establishment of Elder Abuse Fatality Review Teams (EAFRT) [38]. Possible members of these teams include not only lawyers but also forensic scientists, gerontologists and mental health workers. The ABA investigates and verifies the causes of death in cases that they suspect either involve or cause elder abuse, in order to improve a system that has failed to prevent abuse as the cause or primary factor of death and to provide services that lead to prevention [39]. Establishing a new institution and system for the verification of fatal cases caused by elder abuse may be needed in Japan too.

From a global point of view, a better understanding of prevention is regarded as important in elder abuse [13], less social support is known to increase the risk of elder abuse [3], and high social support may reduce the risk of elder abuse [40]. In general, many clients and family members do not know or understand what social resources are available or could help [37]. It appears that both formal and informal social resources available to older adults are important for prevention of elder abuse, and support is needed from related institutions to promote the availability and use of such resources. Developing a comprehensive approach to prevention for elder abuse is still in its early stages in many countries, because resources are scarce and there is no solid evidence on preventing elder abuse [13]. This is consistent with the issues raised in our study about needing more human and financial resources and disseminating policy for prevention of elder abuse.

### 4.3. Current Status and Issues of Cooperation between Hospitals, Municipalities and Public Community General Support Centers

In this study, about 50% of municipalities had collaborated with hospitals in at least one case, mainly sharing information about the victims’ disease status. Indeed, some collaborative efforts seemed effective. However, we also found room for improvement in follow-up work and feedback from municipalities. In Japan, it has been reported that more than 60% of elderly abuse victims who died had previously visited a medical institution [16]. There may have been some issues with collaboration and information-sharing in these cases. It is necessary to establish a system to share the information obtained in cases to prevent further abuse [41], and parties such as municipalities, public community general support centers and outside agencies, similar to the ABA’s EAFRT, should be involved in verifying cases in order to continually improve the system and future services.

In this study, only a relatively small percentage of respondents working in hospitals had collaborated on cases with municipalities. Also, only a small number of hospitals reported that they handled elder abuse cases (16%). Abused older adults are more likely to visit emergency departments than non-abused elderly people [42]. Staff in the emergency department should therefore be mindful of the potential for elder abuse in the cases they manage [43], as it could help medical institutions increase their responsiveness to elder abuse. This requires the cooperation of medical social workers in information gathering and information sharing. In our study, many of the clinical departments that were consulted about abuse were internal medicine or emergency departments. Many staff were reported to be finders or informers of elder abuse at hospitals in this study, suggesting that medical institutions may have opportunities to detect elder abuse. Another study shows that a sense of professional responsibility may facilitate detecting elder abuse in health care institutions [44]. In some emergency departments outside Japan, social workers trained in assessment and intervention for elder abuse perform the first evaluation and then discuss and plan the next steps with other healthcare providers. Their work includes integration of social resources [45]. Leveraging the skills of healthcare providers and social workers to evaluate and respond to needs in elder abuse cases at hospitals may help with more comprehensive service delivery [46]. In reality, however, given that doctors report about only 2% of all cases of suspected elder mistreatment [47], medical experts should actively collaborate with experts in other fields [22]. To facilitate forensic investigation of elder abuse and neglect, Kogan et al. [48] have suggested the development of a tool to properly record the results of physical examinations of injured older adults in medical institutions; however, they also pointed out several problems in this approach that can be burdensome for busy medical professionals. Clearly, there are many issues to address in relation to the current limited resources affecting all institutions.

Although nearly half of the municipalities in this study responded that they had collaborated with hospitals, many municipalities reported that they handled difficult-to-judge cases, so collaboration involving hospitals generally, as well as forensic medicine departments, is important. As an example of collaboration overseas, experts in various fields at the EAFC in Orange Country, California, conduct case meetings every other week [49]. Collaboration between the “client system” (health, social, etc.) and the “judicial system” (law enforcement agencies, lawyers, etc.) is crucial to manage complex cases; however, because they often have different aims, approaches and points of view, the system as a whole is designed to reconcile these differences through such regular meetings [50]. Violence against older adults is a complicated problem and effort and resources across various departments are needed to deal with it [21]. In another example, a state agency and medical schools in Texas have formed a partnership to move the Texas Elder Abuse and Mistreatment (TEAM) project forward. This project involves numerous professionals and institutions representing, for example, law enforcement, mental health and public health, and thus covers all aspects of elder abuse work collaboratively [51]. This could be a good model to follow to encourage collaboration among related institutions dealing with elder abuse and to promote prompt cooperation between experts in different fields.

This study identified the need to provide regular opportunities for exchange of opinions and also inform people about the enquiries desks in order to strengthen cooperation between related institutions, so opportunities for regular interaction between them are clearly needed. In addition, a team including staff from forensic medicine departments and various institutions needs to be established in order to give feedback on and verify cases. A support network is needed in the isolation caused in the COVID-19 pandemic [52]. In this regard, and taking into account the current limited resources, we need to solve the issues of how to hold meetings for efficient exchange of ideas that can bridge differences in member’s positions and how to secure human and financial resources for this from administrative bodies. In order to provide better service to clients, workers need to be trained, and funds are needed to do so [53]. Establishing a research organization comprising specialists from various fields that deal with cases of elder abuse, including fatal cases, may be useful for the prevention of elder abuse in Japan as well as in other countries where systems aimed at such prevention are being developed.

### 4.4. Limitations of the Study

This study involved a limited number of institutions that were randomly selected for inclusion, and therefore the findings should be verified by increasing the sample size in future research. We plan to survey other institutions that may detect cases of elder abuse and, based on the results, contribute to establishing a system that is in line with policy, social welfare, and other current circumstances.

## 5. Conclusions

This study compared cases of elder abuse at forensic medicine departments with those at related institutions and investigated the status of collaboration between them. This is the first study to clarify the forensic characteristics of cases of elder abuse at forensic medicine departments in Japan. More autopsy cases involving elder abuse were reported in our study than cases reported by the government, suggesting that more cases likely exist. For early detection of elder abuse, sharing information about the characteristics of cases is important. Tackling issues of budgetary allocations and informing people about the role that forensic medicine departments can play in managing and preventing elder abuse is needed in order to strengthen collaboration between forensic medicine departments and related institutions. A research organization of experts from different fields may also help in the prevention of elder abuse.

## Figures and Tables

**Table 1:** Sites and types of injury in cases of elder abuse at forensic medicine departments.

Site/Type	n	%	Site/Type	n	%
Head/incised wound	3	21.4	Chest or abdomen/fracture	7	50.0
Head/stab wound	2	14.3	Back/incised wound	2	14.3
Head/impression	1	7.1	Back/stab wound	2	14.3
Head/abrasion	7	50.0	Back/abrasion	6	42.9
Head/subcutaneous haemorrhage	12	85.7	Back/subcutaneous hemorrhage	9	64.3
Head/contusion	4	28.6	Back/burn	3	21.4
Head/laceration	3	21.4	Back/fracture	1	7.1
Head/burn	3	21.4	Upper extremity/incised wound	3	21.4
Head/fracture	1	7.1	Upper extremity/stab wound	3	21.4
Neck/incised wound	4	28.6	Upper extremity/abrasion	4	28.6
Neck/stab wound	4	28.6	Upper extremity/subcutaneous hemorrhage	11	78.6
Neck/impression	7	50.0	Upper extremity/contusion	1	7.1
Neck/abrasion	4	28.6	Upper extremity/laceration	1	7.1
Neck/subcutaneous hemorrhage	9	64.3	Upper extremity/burn	3	21.4
Neck/contusion	1	7.1	Lower extremity/incised wound	2	14.3
Neck/laceration	1	7.1	Lower extremity/stab wound	2	14.3
Neck/burn	3	21.4	Lower extremity/abrasion	5	35.7
Neck/fracture	2	14.3	Lower extremity/subcutaneous hemorrhage	9	64.3
Chest or abdomen/incised wound	4	28.6	Lower extremity/contusion	2	14.3
Chest or abdomen/stab wound	5	35.7	Lower extremity/laceration	1	7.1
Chest or abdomen/abrasion	4	28.6	Lower extremity/burn	3	21.4
Chest or abdomen/subcutaneous hemorrhage	10	71.4	External genital/burn	2	14.3
Chest or abdomen/contusion	1	7.1	Anus/burn	2	14.3
Chest or abdomen/laceration	1	7.1			
Chest or abdomen/burn	3	21.4			
Site	n	%	Type	n	%
Head	13	92.9	Subcutaneous hemorrhage	12	85.7
Neck	12	85.7	Abrasion	10	71.4
Back	12	85.7	Impression	7	50.0
Upper extremities	12	85.7	Fracture	7	50.0
Chest or abdomen	11	78.6	Stab wound	5	35.7
Lower extremities	10	71.4	Contusion	5	35.7
External genitalia	2	14.3	Incised wound	4	28.6
Anus	2	14.3	Laceration	3	21.4
			Burn	3	21.4

**Table 2:** Responses regarding collaboration in elder abuse between forensic medicine departments and related institutions.

Things enabled or possibly enabled by collaborating with hospitals		
	n	%
Assessment from both clinical and forensic perspectives	22	73.3
Accurate judgement about mechanism of injury	18	60.0
Collaboration with clinical departments	17	56.7
Able to avoid overlooking injuries	13	43.3
Collaboration with the manager of the regional medical liaison office in the hospital	7	23.3
Other	3	10.0
Requirements for strengthening collaboration with hospitals		
	n	%
Conducting training on the roles of forensic medicine in measures against elder abuse	18	58.1
Providing information about enquiries desks for handling elder abuse at hospitals and forensic medicine departments	14	45.2
Having regular opportunities to exchange opinions	14	45.2
Preparing a list of forensic medicine departments that can handle elder abuse	10	32.3
Preparing a manual for collaboration with forensic medicine departments in each hospital	10	32.3
Preparing a brochure about the roles of forensic medicine in measures against elder abuse	8	25.8
Other	3	9.7
Things needed for hospitals to easily contact forensic medicine departments		
	n	%
Building relationships for easy consultation	17	54.8
Exchanging opinions between forensic practitioners and hospitals face to face	16	51.6
Clarifying the person in charge and the role of hospitals in elder abuse	12	38.7
Public awareness activities about assessments for elder abuse	11	35.5
Other	6	19.4
Things enabled or possibly enabled by collaborating with municipalities		
	n	%
Precise judgement of whether abuse occurred	11	55.0
Able to provide an objective explanation to the abuser (including suspected abuse)	10	50.0
Precise judgement of whether temporary separation is needed	10	50.0
Useful for court documents	9	45.0
Improvement of the quality and knowledge of staff in municipalities	9	45.0
Other	2	10.0
Requirements for strengthening collaboration with municipalities		
	n	%
Budget acquisition for direct and indirect assessment of living patients	17	53.1
Conducting training on the roles of forensic medicine in measures against elder abuse	15	46.9
Having regular opportunities to exchange opinions	15	46.9
Providing information about enquiries desks for handling elder abuse in municipalities and forensic medicine departments	14	43.8
Preparing a list of forensic medicine departments that can handle elder abuse	13	40.6
Preparing a brochure about the roles of forensic medicine in measures against elder abuse	9	28.1
Other	4	12.5
Things needed for municipalities to easily contact forensic medicine departments		
	n	%
Exchanging opinions between forensic practitioners and municipalities face to face	23	71.9
Clarifying the person in charge and the role of municipalities in elder abuse	15	46.9
Building relationships for easy consultation	13	40.6
Public awareness activity about assessment of elder abuse	12	37.5
Other	3	9.4

**Table 3:** Cases of elder abuse at related institutions.

	Hospitals	Municipalities	Public Community General Support Centers
Case of elder abuse in FY2019	Yes (16%) No (84%)	Yes (81.5%) No (18.5%)	Yes (87%) No (13%)
Sex	Male 21 Female 49	Male 398 Female 1155	Male 209 Female 532
Age	80s (38%) 70s (35%)	80s (42%) 70s (34%)	80s (49%) 70s (32%)
Type of abuse	Physical abuse (40%) Neglect (33%)	Physical abuse (46%) Neglect (13%)	Physical abuse (49%) Neglect (13%)
Perpetrator	Son (39%) husband (24%)	Son (40%) Husband (23%)	Son (42%) Husband (20%)
Location of abuse	Home (95.5%)	Home (84%)	Home (97%)
Means of abuse	Beating or kicking (42%) Pushing (14%)	Beating or kicking (62%) Pushing (6%)	Beating or kicking (68%) Pushing (6%)
Reporter of abuse	Care manager (63.6%) Hospital staff (45.5%)	Care manager (72.3%) Police (55.9%)	Care manager (77.9%) Police (40.7%)
Background of elder abuse	Staff noticed findings of abuse (including suspected abuse) during medical examination (60.9%)	-	-
Cases reported to municipalities	Yes (60.9%) No (39.1%)	-	-
Received feedback from municipalities	Yes (53.8%) No (46.2%)	-	-
Route of consultation	Public community general support centers refer the patient (47.8%) Emergency transport (43.5%)	-	-
Clinical department consulted about abuse	Internal medicine (68.2%) Emergency (40.9%)	-	-
Case in which the victim was temporarily separated from the perpetrator	-	Yes (78.7%) No (21.3%)	Yes (66.2%) No (33.8%)
Case in which could not make a decision on temporary separation	-	Yes (28.4%) No (71.6%)	Yes (29.1%) No (70.9%)
Reason a decision could not be made on temporary separation	-	Difficulty judging whether the injury was abuse or an accident (35.4%)	Difficulty judging whether the injury was abuse or an accident (21.8%)
Follow-up work on cases	-	No (32.8%)	No (13.2%)
Case where it was difficult to judge whether abuse occurred	Yes (47.6%) No (52.4%)	Yes (49.2%) No (50.8%)	Yes (42.3%) No (57.7%)
Reason it was difficult to judge whether abuse occurred	Difficulty judging whether neglect occurred Difficulty distinguishing between abuse and repeated falls at home	Because the victim had dementia and an interview was not possible, so staff could not judge if caused by fall or violence	Because the victim with fall risk had dementia and did not remember, so it was difficult to judge if abuse or fall caused the bruising
Trends noticed in elder abuse	Elder abuse definitely increased (65%)	Elder abuse definitely increased (41.3%)	Elder abuse definitely increased (49.5%)
Photos taken of injury	Yes (68.2%)	Yes (64.5%)	Yes (60%)
Observation of injury	-	Visual observation (92.5%) Photos taken beforehand (54%)	Visual observation (92.1%) Photos taken beforehand (42.9%)

**Table 4:** Responses on collaboration between hospitals, municipalities and public community general support centers.

	Hospitals		Municipalities		Public Community General Support Centers	
Effects of Collaboration	n	%	n	%	n	%
Precise judgment of whether abuse occurred	15	68.2	79	68.7	48	46.2
Precise judgement of whether temporary separation is needed	13	59.1	82	71.3	62	59.6
Able to provide objective explanations to the abuser	8	36.4	75	65.2	65	62.5
Useful for court documents	2	9.1	11	9.6	10	9.6
Improvement of the quality and knowledge of staff	5	22.7	33	28.7	23	22.1
Leading to support for handing abuse	18	81.8	69	60.0	59	56.7
Other	0	0	7	6.1	12	11.5
Improvements for strengthening collaboration	n	%	n	%	n	%
Conducting training on collaboration	65	48.5	118	52.7	120	52.9
Creating manuals for collaboration	69	51.5	100	44.6	97	42.7
Providing information about enquiries desks	74	55.2	93	41.5	90	39.6
Having opportunities for regular exchange of opinions	67	50.0	137	61.2	134	59.0
Other	4	3.0	6	2.7	10	4.4
Reasons for lack of collaboration with forensic practitioners	n	%	n	%	n	%
There was no precedent	90	64.7	114	48.7	140	57.1
There was no forensic practitioner nearby	49	35.3	165	70.5	165	67.3
Not understanding how forensic medicine is involved in elder abuse cases	59	42.4	159	67.9	179	73.1
Not knowing about the enquiries desk for consultation	51	36.7	127	54.3	157	64.1
Able to handle cases of elder abuse with hospitals	13	9.4	65	27.8	55	22.4
No budget available	4	2.9	63	26.9	33	13.5
Other	7	5.0	7	3.0	16	6.5
Improvements for strengthening collaboration with forensic practitioners	n	%	n	%	n	%
Conducting training on the roles of forensic medicine in measures against elder abuse	60	47.2	127	62.0	154	73.0
Creating brochures about the roles of forensic medicine in measures against elder abuse	72	56.7	97	47.3	116	55.0
Providing information about enquiries desks	76	59.8	83	40.5	102	48.3
Preparing a list of departments of forensic medicine departments that can handle elder abuse	33	26.0	94	45.9	95	45.0
Securing a budget for assessment by a forensic practitioner	22	17.3	42	20.5	50	23.7
Having opportunities to regularly exchange opinions	34	26.8	62	30.2	80	37.9
Other	3	2.4	20	9.8	16	7.6
Things needed for future collaboration with forensic practitioners	n	%	n	%	n	%
Building relationships for easy consultation	84	66.1	147	64.2	145	61.2
Clarifying the person in charge and the role of related institutions in handling elder abuse	56	44.1	86	37.6	69	29.1
Exchanging opinions face to face	46	36.2	129	56.3	139	58.6
Public awareness activities about assessments for elder abuse	41	32.3	69	30.1	76	32.1
Other	3	2.4	17	7.4	29	12.2

## Data Availability

The data that support the findings of this study are available from the corresponding author upon reasonable request.
